# The Use of Chat-GPT 5.2 by Patients Affected by Rotator Cuff Tears Leads to Inaccurate Diagnosis and Treatment Suggestions: A Study by SICSeG (Italian Society of Shoulder and Elbow Surgery)

**DOI:** 10.3390/jcm15103878

**Published:** 2026-05-18

**Authors:** Roberto de Giovanni, Edoardo Gaj, Luciano Mottola, Antonio Benedetto Cecere, Martina Coppola, Raffaele Garofalo, Andrea Cozzolino

**Affiliations:** 1 UOC Orthopaedic and Trauma Surgery, Department of Public Health, Federico II University Hospital, Via Pansini 5 Building 12, 80131 Naples, Campania, Italy; andrea.cozzolino@hotmail.it; 2 Ospedale Israelitico di Roma, 00148 Rome, Lazio, Italy; edoardogaj@gmail.com; 3 P.O. “S. Giuseppe Moscati” di Aversa, 81031 Caserta, Campania, Italy; luciano.mottola12@gmail.com; 4 P.O. Giugliano, AslNapoli2Nord, 80014 Naples, Campania, Italy; antoniobenedettocecere@gmail.com; 5 AORN Ospedale dei Colli, C.T.O., 80131 Naples, Campania, Italy; martinacoppola_96@virgilio.it; 6 Humanitas Research Hospital, 20089 Milan, Lombardia, Italy; raffaelegarofalo@gmail.com

**Keywords:** rotator cuff tear, artificial intelligence

## Abstract

**Background:** Patients increasingly rely on freely available artificial intelligence tools, such as Chat-GPT, to obtain diagnostic and therapeutic information for different orthopedic conditions. While preliminary studies have evaluated its educational potential, evidence regarding its diagnostic accuracy and treatment recommendations in rotator cuff pathology remains limited. This study aimed to assess the ability of Chat-GPT 5.2 to correctly diagnose rotator cuff tears and propose treatment strategies, comparing its performance with that of expert shoulder surgeons. **Materials and Methods:** Five clinical cases representing common rotator cuff tear patterns were retrospectively selected in an exploratory pilot analysis. For each case, MRI images, radiologic reports, and clinical information were sequentially provided to Chat-GPT 5.2 using standardized prompts simulating a patient inquiry. Responses were compared with those of three experienced shoulder surgeons. Diagnostic accuracy, treatment recommendations, postoperative management suggestions, and complication descriptions were analyzed. Krippendorff’s alpha was used to assess interobserver agreement. **Results:** Chat-GPT 5.2 did not correctly diagnose any case using MRI images alone, whereas human examiners reached accurate diagnosis in most cases. Treatment recommendations provided by Chat-GPT were non-specific when based solely on imaging or radiologic reports, but became more defined after a detailed lesion description. Interobserver agreement between Chat-GPT and surgeons regarding treatment was inconsistent across cases. Postoperative rehabilitation advice and complication descriptions were accurate and comprehensive, demonstrating high consistency with published data. **Conclusions:** The results of this pilot analysis suggest that the use of Chat-GPT 5.2 by patients affected by rotator cuff tears could potentially lead to misdiagnosis and wrong treatment advice, while on the contrary it could be used by physicians to better illustrate postoperative protocols, complications and expected outcomes.

## 1. Introduction

The diffusion of artificial intelligence (AI) and its free availability to the general population through large language models (LLMs) is modifying the doctor–patient relationship and taking the medical field by storm [1]. Chat Generative Pretrained Transformer (Chat-GPT) is one of the most widely used AI programs available online, and it has demonstrated the ability to improve the readability of the scientific literature [2], and to answer patient questions about various shoulder and orthopedic conditions [3,4,5]. Preliminary evaluations in shoulder surgery show that Chat-GPT can provide generally accurate and patient-friendly educational information; however, its responses often lack up-to-date evidence and do not match the specificity or reliability of an orthopedic surgeon’s expertise [6]. In pediatric orthopedics, large language models often align with established clinical guidelines, yet they continue to produce a substantial number of incomplete or inaccurate recommendations, highlighting persistent limitations in reliability and transparency [7]. The accuracy of self-diagnosis using this type of AI has been questioned by several authors, both in simple situations (such as distal radius fractures or common orthopedic diseases [8,9]) and in more complex ones (like discal herniation and combined elbow injuries [10,11]).

The accuracy of Chat-GPT in shoulder pathology has recently been investigated [12,13,14] but framing AI-generated answers in daily practice has its challenges. Lacking guidelines, a theoretically correct model or universal correct answer should account for multiple tear-related and patient-related variables. In rotator cuff pathology, the controversy about different treatment options according to the wide variability of lesion patterns has sparked debate among shoulder surgeons for decades [15], due to important differences between degenerative [16] and traumatic tears [17]. Management strategies based on patient-dependent factors even in the presence of similar lesions, like activity level, main complaint and functional requests, are further elements of controversy [18,19]. To this day, the timing of intervention [20], type of treatment [21] and surgical technical aspects [22] remain subjects of round table discussion among experts [23].

Furthermore, failures [24,25] and complications have been described in long-term follow-up studies and represent crucial information that should be communicated to the patients in order to give them realistic expectations and ensure optimal compliance in the postoperative period; for instance, the weight of a timely rehabilitation has been proven to be systematically underestimated [26].

The aim of this study was to exploratorily investigate the diagnostic accuracy and therapeutic recommendations provided by the mainstream LLM Chat-GPT, comparing its performance with that of a group of expert shoulder surgeons.

## 2. Materials and Methods

The study was conceived and conducted by the Innovation Committee of the Italian Shoulder and Elbow Society—S.I.C.S.eG. (*Società Italiana di Chirurgia di Spalla e Gomito*)—with the involvement of the Society’s Directive Committee. Given the preliminary nature of the investigation, the study was designed as an exploratory pilot analysis with a feasibility-oriented design. This approach was chosen to reflect both the exploratory aim of evaluating the responses generated by Chat-GPT 5.2 in selected clinical scenarios and the need to assess whether this type of structured evaluation could represent a feasible model for future, larger investigations.

**Figure 1:**
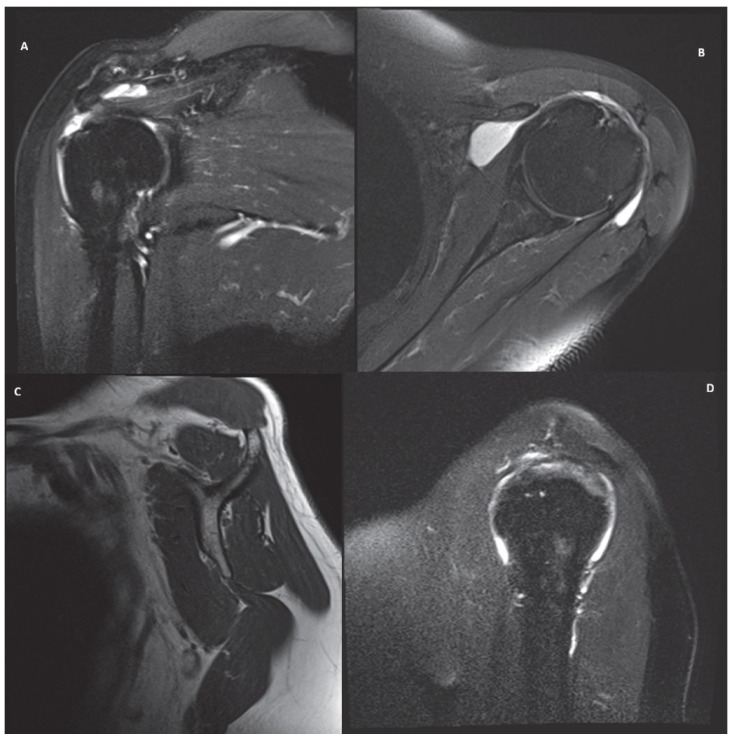
MRI images of a partial bursal supraspinatus tear in coronal (**A**), axial (**B**) and sagittal (**C**,**D**) view.

**Figure 2:**
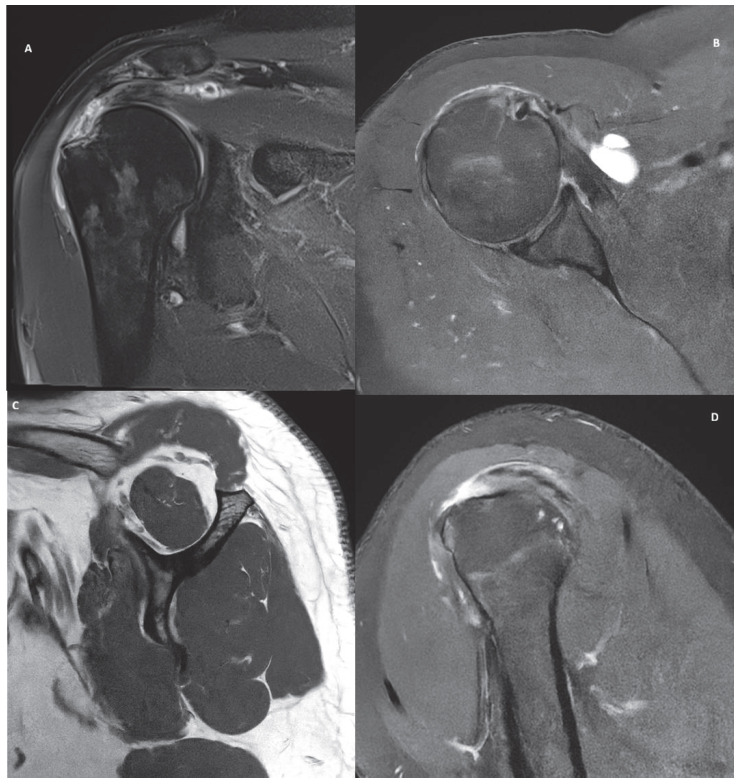
MRI images of an isolated supraspinatus full thickness tear in coronal (**A**), axial (**B**) and sagittal (**C**,**D**) view.

**Figure 3:**
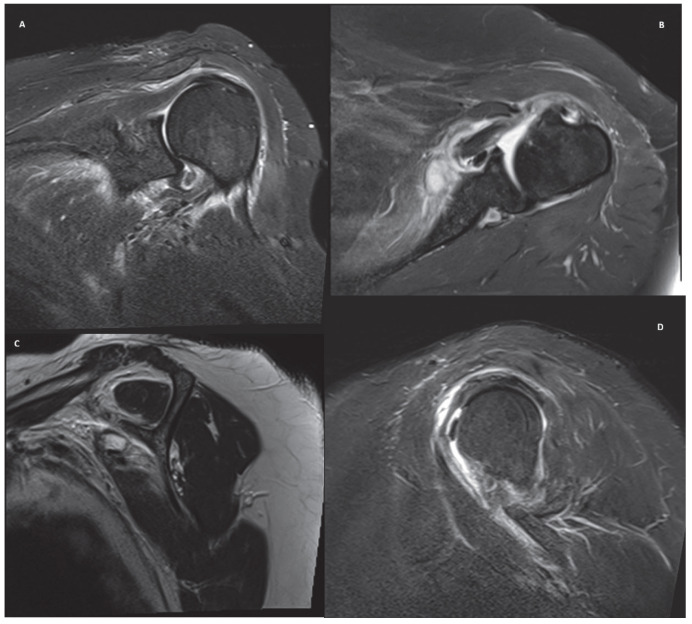
MRI images of an isolated Lafosse type 2 subscapularis tear in coronal (**A**), axial (**B**) and sagittal (**C**,**D**) view.

**Figure 4:**
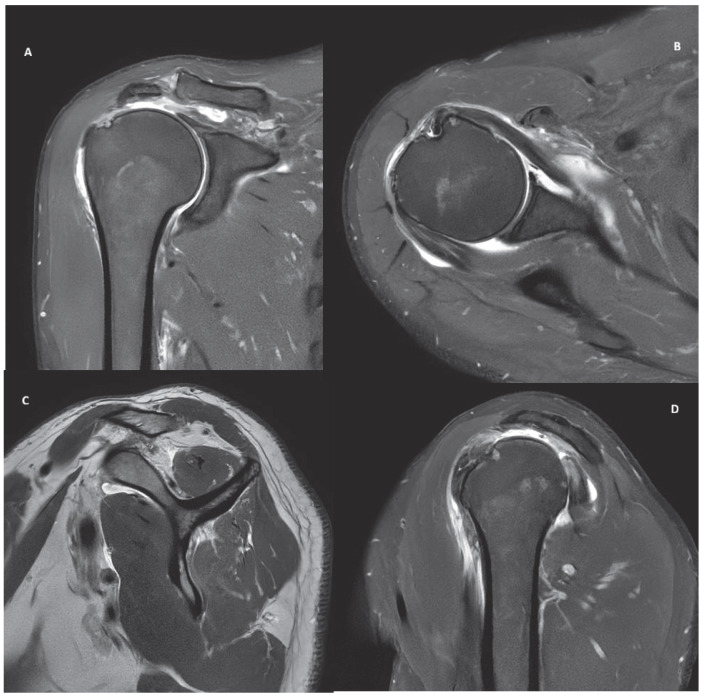
MRI images of a posterosuperior massive rotator cuff tear involving both supraspinatus and infraspinatus tendons in coronal (**A**), axial (**B**) and sagittal (**C**,**D**) view.

**Figure 5:**
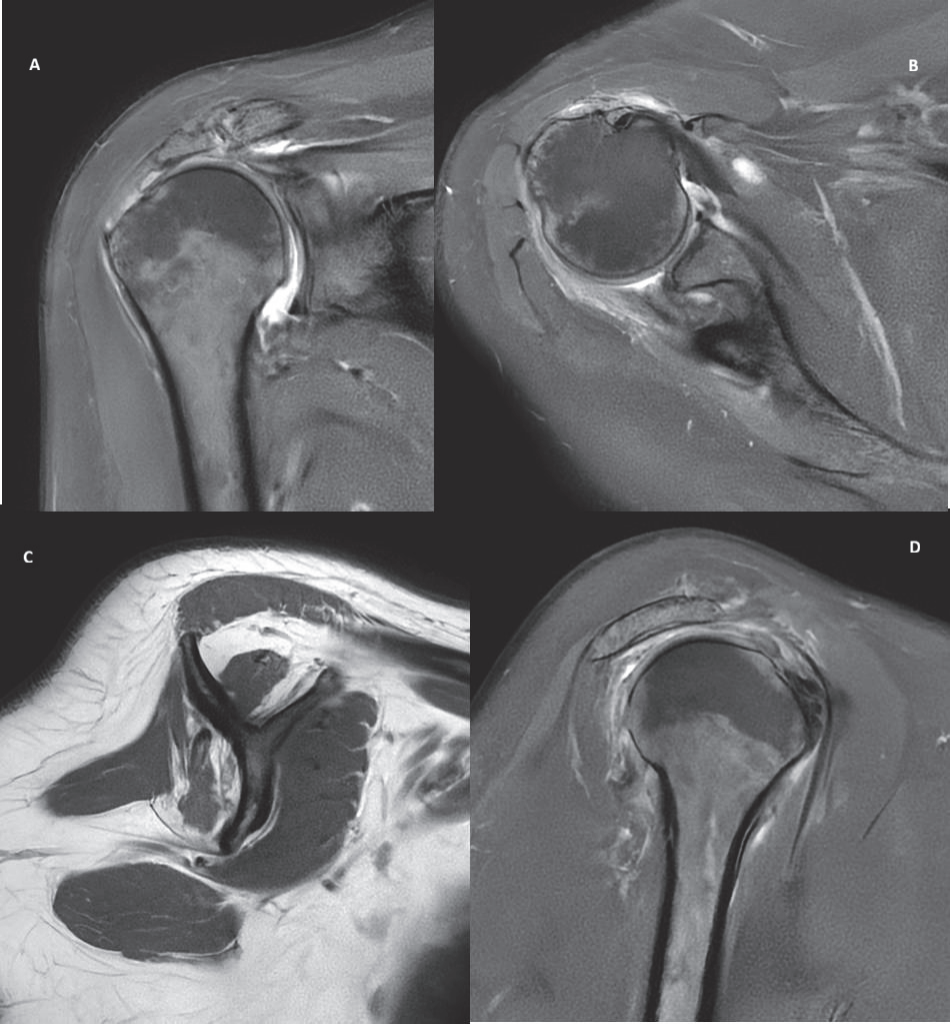
MRI images of an irreparable posterosuperior rotator cuff tear with supraspinatus, infraspinatus involvement and Fuchs > 3 fat atrophy in coronal (**A**), axial (**B**) and sagittal (**C**,**D**) view.

Five cases, representing common clinical scenarios encountered in the diagnosis and treatment of rotator cuff tears, were retrospectively selected and included in the study:

1. Partial bursal-sided supraspinatus tear (Figure 1).
2. Isolated full-thickness supraspinatus tear (Figure 2).
3. Isolated Lafosse type 2 subscapularis tear (Figure 3).
4. Posterosuperior massive rotator cuff tear involving both supraspinatus and infraspinatus tendons (Figure 4).
5. Irreparable rotator cuff tear with supraspinatus, infraspinatus and subscapularis involvement and massive fat atrophy (Figure 5).

Clinical data (such as age, occupation and activity level, type of sport or recreational activity, VAS for pain, Constant score, duration of symptoms, previous treatments, physical examination including passive and active range of motion, and rotator cuff tests) and MRI DICOM files for each case were collected. A high-resolution 1.5 Tesla MRI, using a standard shoulder protocol, was obtained in all cases, and the four most representative images (anonymous JPEG files) were selected. Specifically, the following cuts were considered necessary to identify and characterize the lesion type: a coronal T2 fat-sat slice at the level of the anterior facet of the greater tuberosity; a parasagittal T2 fat-sat slice at the level of the greater tuberosity; a parasagittal T1 slice at the insertion of the spine on the scapula body; and, an axial T2 fat-sat slice under the coracoid tip. Chat-GPT 5.2 was used through a private connection to prevent further AI training. A specific set of questions was repeated for each scenario, and a new connection was established for each question after clearing the chat history. To determine the most appropriate prompt to use, we first asked the same LLM which question would be optimal for developing our study.

To evaluate the diagnostic ability of the AI, a set of questions were defined and the following prompt was used: “You are an orthopedic surgeon expert in shoulder pathology. Evaluate the following uploaded shoulder MRI images and define the correct diagnosis”. In a second step, the radiologist’s report was added in the prompt, followed by the request: “could you please clearly and concisely define my diagnosis?”

Finally, the therapeutic recommendations produced by Chat-GPT 5.2 were assessed using the following prompt: “You are an orthopedic surgeon expert in shoulder pathology. Given the presence of a [diagnosis of each of the five cases] which treatment do you recommend?”

To evaluate Chat-GPT 5.2’s ability to provide an appropriate treatment plan, simulating how a patient might use the tool, we first provided only the radiological report. In cases with inconclusive responses, we sequentially added clinical information: patient age, pain level, duration of symptoms, occupational and recreational activity level, and comorbidities. To avoid the influence of subjective radiologic interpretation, we then provided a synthetic diagnosis including rotator cuff tear type, tear size on parasagittal image (defined as <1 cm, between 1 and 2 cm, and >2 cm), tendon retraction in the coronal plane according to Patte classification, and muscle fatty degeneration according to Fuchs [27]. Again, in the case of inconclusive answers, clinical data were added following the same sequence (patient age, work type and level, pain, duration of symptoms, comorbidities, clinical examination). Finally, we asked Chat-GPT to describe postoperative complication types and rates, as well as the postoperative management plan. All answers were recorded.

Chat-GPT 5.2’s responses were compared with those of a panel of three experienced surgeons (Executive Committee of SICSEG) to evaluate interobserver agreement.

### Statistical Analysis

Continuous data were reported as mean or as numbers (percentages) ± standard deviation (SD). The distribution of variables was tested using the Shapiro–Wilk test. Comparisons between study and control groups were performed using Student’s *t*-test for normally distributed continuous variables, Wilcoxon rank-sum test for non-normally distributed continuous variables, and chi-square for categorical variables.

The diagnostic evaluation of both Chat-GPT 5.2 and the human control group was resumed in a dichotomous variable (correct/incorrect), based on intraoperative findings.

Treatment type was defined as conservative, surgical or not defined. The amount of clinical information required by the AI and the human control group to formulate a therapeutic recommendation was also recorded. Interobserver agreement for both diagnostic and therapeutic evaluation was quantified using Krippendorff’s alpha scores. Strength of agreement for alpha (α) values was interpreted according to Krippendorff’s scale: an α ≥ 0.800 indicates strong reliability; between 0.667 and 0.800 indicates low reliability; <0.667 indicates really low reliability. Complication types and rates described by both the AI and the human control group were compared to the data reported in the literature [28,29].

## 3. Results

### 3.1. Diagnostic Ability

In none of the included cases was Chat-GPT 5.2 able to identify the correct diagnosis using MRI images alone. On the contrary, the three independent human examiners provided the same correct diagnosis in four out five cases (Table 1). Interobserver agreement among the three experienced surgeons was strong for tendon retraction (α = 0.85) whereas agreement was lower for muscle fatty infiltration and tear size evaluation (α = 0.75 and 0.7, respectively).

### 3.2. Treatment Recommendation

The use of the radiologic report allowed Chat-GPT 5.2 to finally recognize the presence of the rotator cuff tear. However, the AI was unable to provide a specific therapeutic recommendation based only on the imaging report, and even after receiving all available clinical information, both conservative and surgical treatment possibilities were proposed by the LLM. On the contrary, the use of a detailed description of the rotator cuff tear, including tear size, tendon retraction, and degree of muscular fatty infiltration, let Chat-GPT 5.2 propose a specific therapeutic plan without requiring additional clinical information in cases 1, 3 and 4. For cases 2 and 5, the AI requested further data regarding age and activity level. The human control group reported that the radiologic report alone was not useful for treatment decision-making. A definitive treatment indication was formulated only after incorporating at least two clinical variables (age and pain level) for the subscapularis tear and massive posterosuperior tear (cases 3 and 4). For the partial tear, isolated supraspinatus tear and irreparable rotator cuff tear (cases 1, 2 and 5), two of the three surgeons provided the final therapeutic recommendation only after considering all available clinical information. Low interobserver agreement was found between Chat-GPT 5.2 and the three experienced surgeons regarding conservative and surgical treatment recommendations for case 2, 4 and 5 (α = 0.65, 0.38 and 0.65, respectively). Conversely, perfect agreement favoring surgical treatment was found for cases 1 and 3 (α = 1). Treatment indications are summarized in Table 2.

### 3.3. Postoperative Rehabilitation, Complication Rate and Types

Postoperative rehabilitation protocols suggested by both the AI and the human control group were very similar. In the case of rotator cuff repair, both recommended the use of a brace for 4–6 weeks, followed by physiotherapy with a trained therapist 2–3 times per week. Rehabilitation was consistently divided into two phases: an initial phase focused on restoring the range of motion, followed by a strengthening program. Chat-GPT 5.2 provided consistent and comprehensive descriptions of postoperative complication types and rates (Table 3), whereas the human control group primarily reported re-tear and infection as the main postoperative complications.

## 4. Discussion

### 4.1. Rotator Cuff Tear Diagnosis

Our results showed that the ability of Chat-GPT 5.2 to identify rotator cuff tears on both MRI images and radiologist reports is poor. Recent advances in artificial intelligence, particularly deep learning and the implementation of Agents able to autonomously execute even more complex tasks, have demonstrated promising potential in reducing diagnostic errors in musculoskeletal imaging [30].

Previous studies evaluating the diagnostic ability of different deep learning models on rotator cuff tears observed an accuracy ranging between 70% and 100%, with high sensitivity and specificity [31], and similar analyses in terms of the reliability of LLMs in challenging clinical settings are being increasingly published [32]. Although future improvements in generative AI may enhance the diagnostic performance of freely available tools, the transition from Chat-GPT 4 to Chat-GPT 5 has not meaningfully addressed this limitation. Moreover, the generative AI tested in our study did not provide vague or cautious statements; rather, it frequently generated specific but incorrect diagnoses, which could create confusion and erode patient trust as already highlighted in previous research [33]. Given the increasing number of patients affected by musculoskeletal diseases, relying on AI for accurate, readable, and condition-specific information [34], it is reasonable to foresee in the future a rise in second opinion requests and medicolegal concerns associated with AI-generated misinformation [34,35].

Human radiologic reports remain the cornerstone of diagnostic decision-making in rotator cuff pathology. These reports, however, are a subjective description of an image and its quality is strongly dependent on the radiologist’s ability and interpretation. Moreover, despite published recommendations, MRI reports often lack essential details such as tear retraction, size and muscle atrophy, providing only general morphological descriptions [36]. In our study, an inaccurate MRI report analyzed by Chat-GPT led to inconclusive outputs in all the cases, whereas the specific tear description allowed the AI to generate more consistent therapeutic suggestions. These findings underline that high-quality data are essential for reliable AI performance, while vague or incomplete information can easily produce misleading or harmful responses. This aspect is especially relevant given that the patient-driven use of the LLM is not expected to be pitch-perfect in terms of the clarity of the complaint and accuracy of the description.

### 4.2. Rotator Cuff Tear Treatment

The indication for a specific type of treatment in patients affected by rotator cuff tears remains controversial and continues to represent one of the most debated aspects of shoulder surgery [25]. Although rotator cuff repair is a widely performed procedure, the mere presence of a tendon tear does not automatically constitute an indication for surgery. Most studies agree that surgical intervention should be proposed only in selected cases, particularly in patients presenting with persistent, functionally limiting, and disabling symptoms that do not improve after an adequate and properly conducted course of conservative management. In addition, the patient’s functional demands, occupational requirements, age, comorbidities, activity level, and expectations should all be carefully considered before recommending operative treatment. Indeed, several studies, including investigations with long-term follow-up, have demonstrated that conservative treatment can achieve outcomes comparable to those of surgical treatment in selected patients. These findings suggest that non-operative management should not be considered merely as a temporary solution or a second-line option, but rather as a valid therapeutic strategy in appropriately selected cases. Among the factors associated with a favorable response to conservative treatment, one of the most relevant appears to be the patient’s expectation regarding the efficacy of physical therapy [36]. This aspect is particularly important because it highlights the role of patient education, adherence to rehabilitation, and confidence in the proposed treatment pathway. Therefore, there is no universally correct or standardized answer to the question “should my rotator cuff tear be repaired?”. The final decision must be individualized and should take into account both lesion-related variables and patient-related variables. Tear size, tendon retraction, muscle fatty infiltration, chronicity, traumatic or degenerative onset, and the presence of associated shoulder disorders must be interpreted together with the patient’s symptoms, functional impairment, clinical examination, general health status, and personal expectations. Moreover, treatment indication should always be discussed directly with the patient in order to ensure a shared decision-making process, avoiding both overtreatment and inappropriate reassurance. In our evaluation, Chat-GPT 5.2 was generally non-specific in suggesting therapeutic options when the diagnosis was unclear to the AI system itself. Because it is unable to independently interpret MRI images or accurately analyze radiology reports, Chat-GPT did not provide a specific treatment recommendation even after all clinical information had been supplied. This limitation is clinically relevant, since imaging findings are central to the characterization of rotator cuff tears and to the formulation of a reliable treatment strategy. On the contrary, when detailed tear characteristics were explicitly described in the prompt, Chat-GPT tended to recommend surgical repair even in the absence of additional clinical information regarding symptoms, functional limitation, previous conservative treatment, or patient-specific factors. For these reasons, we suggest that individuals affected by shoulder pain should not rely on AI systems to determine either the diagnosis or the most appropriate treatment strategy. While AI may provide general educational information, it cannot replace clinical evaluation, imaging interpretation, specialist judgment, and the shared decision-making process between the physician and patient, as of yet.

### 4.3. Complications and Postoperative Management

The AI was at least as specific and articulate as the included orthopedic surgeons in outlining postoperative protocols, complication rates, and types. Therefore, Chat-GPT 5.2 could represent a potentially useful tool for illustrating postoperative rehabilitation, expected outcomes, and possible complications to patients affected by a rotator cuff tear, once the correct diagnosis and treatment strategy have already been established.

Previous studies have demonstrated that re-tear, stiffness and infections are among the most frequent complications after arthroscopic rotator cuff repair; however, the incidence of these events is strictly correlated with follow-up time and tear size [27,37].

The complication rates reported by Chat-GPT 5.2 were largely consistent with those described in the literature, whereas the orthopedic surgeons included in the study generally did not mention specific complication rates or associated risk factors. This may be related to the fact that surgeons often spend most of the consultation discussing treatment options, may feel less confident describing the complete spectrum of complications due to their relatively low incidence in this type of surgery, or may wish to avoid creating excessive concern in the patient. Moreover, it has already been shown that the expectations of patients undergoing rotator cuff repair differ from those of the surgeons [38]; thus, AI could be integrated in the pretreatment interview to obtain better patient and surgeon satisfaction.

## 5. Conclusions

This pilot analysis suggests that the use of Chat-GPT 5.2 by patients affected by rotator cuff tears could potentially lead to misdiagnosis and inappropriate treatment suggestions, particularly when the clinical information provided is incomplete, imprecise, or not supported by a specialist examination and adequate imaging interpretation. In this context, patients may receive overly generic or misleading answers, which could either underestimate the severity of the condition or, conversely, encourage unnecessary concern and inappropriate therapeutic decisions. On the contrary, Chat-GPT 5.2 may represent a useful supportive tool for physicians when used within a controlled clinical setting and under specialist supervision. In particular, it could help clinicians better illustrate postoperative protocols, rehabilitation timelines, possible complications, expected recovery, and overall prognosis in a more accessible and patient-friendly language. Therefore, while its direct use by patients for diagnostic or therapeutic decision-making should be discouraged, its physician-guided application may improve patient education, communication, and understanding of the treatment pathway.

## Figures and Tables

**Table 1:** Diagnosis of each lesion as identified by surgeons and AI.

	Surgeon 1	Surgeon 2	Surgeon 3	Chat-GPT
Bursal-side SPS partial tear	SPS partial-thickness tear	SPS partial-thickness tear	SPS full-thickness tear	SPS full-thickness tear
Full-thickness SPS tear	SPS full-thickness tear	SPS full-thickness tear	SPS full-thickness tear	Anterior paralabral cyst
SSC Lafosse 3 tear	SSC Lafosse 3 tear	SSC Lafosse 2 tear	SSC Lafosse 3 tear	Supraspinatus full-thickness tear
Massive posterosuperior tear	Massive posterosuperior tear	Massive posterosuperior tear	Massive posterosuperior tear	SPS full-thickness tear
Irreparable posterosuperior tear	Irreparable posterosuperior tear	Irreparable posterosuperior tear	Irreparable posterosuperior tear	Retracted chronic SPS full-thickness tear

**Table 2:** Treatment indications as expressed by surgeons and AI.

	Surgeon 1	Surgeon 2	Surgeon 3	Chat-GPT
Bursal side > 50% supraspinatus partial tear	surgical	surgical	surgical	surgical
Full-thickness supraspinatus tear	surgical	surgical	conservative	surgical for “young and active”
Subscapularis tear	surgical	surgical	surgical	surgical
Massive posterolateral tear	surgical	conservative	conservative	surgical
Irreparable tear	conservative	conservative	conservative	surgical according to age and symptoms

**Table 3:** Complications and corresponding rates as reported by AI.

Complication	Incidence	Notes
Re-tear	15–40%	Higher in large tears, older age
Stiffness	5–15%	More in diabetics, females
Infection	<0.5%	Very rare in arthroscopy
Nerve injury	<1%	Usually transient
Deltoid detachment/fracture	<0.5%	Rare, technical issue
DVT/PE	<1%	Very rare in shoulder surgery
Persistent pain/dysfunction	5–15%	May need further management

## Data Availability

All data can be made available through direct contact with the corresponding author.

## References

[1] Montanari Vergallo G., Campanozzi L.L., Gulino M., Bassis L., Ricci P., Zaami S., Marinelli S., Tambone V., Frati P. (2025). How Could Artificial Intelligence Change the Doctor-Patient Relationship? A Medical Ethics Perspective. Healthcare.

[2] Chandra K., Ghilzai U., Lawand J., Ghali A., Fiedler B., Ahmed A.S. (2025). Improving readability of shoulder and elbow surgery online patient education material with Chat GPT (Chat Generative Pretrained Transformer) 4. J. Shoulder Elb. Surg..

[3] Unadkat K.D., Abdulwadood I., Hiredesai A.N., Howlett C.P., Geldmaker L.E., Noland S.S. (2025). ChatGPT 4.0’s efficacy in the self-diagnosis of non-traumatic hand conditions. J. Hand Microsurg..

[4] White C.A., Masturov Y.A., Haunschild E., Michaelson E., Shukla D.R., Cagle P.J. (2025). Can ChatGPT reliably answer the most common patient questions regarding total shoulder arthroplasty?. J. Shoulder Elb. Surg..

[5] Pirkle S., Yang J.W., Blumberg T.J. (2025). Do ChatGPT and Gemini Provide Appropriate Recommendations for Pediatric Orthopaedic Conditions?. J. Pediatr. Orthop..

[6] Kunze K.N., Varady N.H., Mazzucco M., Lu A.Z., Chahla J., Martin R.K., Ranawat A.S., Pearle A.D., Williams R.J. (2025). The Large Language Model ChatGPT-4 Exhibits Excellent Triage Capabilities and Diagnostic Performance for Patients Presenting with Various Causes of Knee Pain. Arthroscopy.

[7] Mika A.P., Mulvey H.E., Engstrom S.M., Polkowski G.G., Martin J.R., Wilson J.M. (2024). Can ChatGPT Answer Patient Questions Regarding Total Knee Arthroplasty?. J. Knee Surg..

[8] Kuroiwa T., Sarcon A., Ibara T., Yamada E., Yamamoto A., Tsukamoto K., Fujita K. (2023). The Potential of ChatGPT as a Self-Diagnostic Tool in Common Orthopedic Diseases: Exploratory Study. J. Med. Internet Res..

[9] Mohamed K.S., Yu A., Schroen C.A., Duey A., Hong J., Yu R., Etigunta S., Kator J., Rhee H.S., Hausman M.R. (2025). Comparing AAOS appropriate use criteria with ChatGPT-4o recommendations on treating distal radius fractures. Hand Surg. Rehabil..

[10] Mejia M.R., Arroyave J.S., Saturno M., Ndjonko L.C.M., Zaidat B., Rajjoub R., Ahmed W., Zapolsky I., Cho S.K. (2024). Use of ChatGPT for Determining Clinical and Surgical Treatment of Lumbar Disc Herniation with Radiculopathy: A North American Spine Society Guideline Comparison. Neurospine.

[11] Nieves-Lopez B., Bechtle A.R., Traverse J., Klifto C., Schoch B.S., Aziz K.T. (2025). Evaluating the Evolution of ChatGPT as an Information Resource in Shoulder and Elbow Surgery. Orthopedics.

[12] Fiedler B., Azua E.N., Phillips T., Ahmed A.S. (2024). ChatGPT performance on the American Shoulder and Elbow Surgeons maintenance of certification exam. J. Shoulder Elb. Surg..

[13] Tashjian R.Z. (2012). Epidemiology, Natural History, and Indications for Treatment of Rotator Cuff Tears. Clin. Sports Med..

[14] Jain N.B., Khazzam M.S. (2024). Degenerative Rotator-Cuff Disorders. N. Engl. J. Med..

[15] Mall N.A., Lee A.S., Chahal J., Sherman S.L., Romeo A.A., Verma N.N., Cole B.J. (2013). An evidenced-based examination of the epidemiology and outcomes of traumatic rotator cuff tears. Arthrosc. J. Arthrosc. Relat. Surg..

[16] Keener J.D., Aleem A.W., Chamberlain A.M., Sefko J., Steger-May K. (2020). Factors associated with choice for surgery in newly symptomatic degenerative rotator cuff tears: A prospective cohort evaluation. J. Shoulder Elb. Surg..

[17] Dunn W.R., Kuhn J.E., Sanders R., An Q., Baumgarten K.M., Bishop J.Y., Brophy R.H., Carey J.L., Holloway G.B., Jones G.L. (2014). Symptoms of pain do not correlate with rotator cuff tear severity: A cross-sectional study of 393 patients with a symptomatic atraumatic full-thickness rotator cuff tear. J. Bone Jt. Surg..

[18] Duncan N.S., Booker S.J., Gooding B.W.T., Geoghegan J., Wallace W.A., Manning P.A. (2015). Surgery within 6 months of an acute rotator cuff tear significantly improves outcome. J. Shoulder Elb. Surg..

[19] Ó Conaire E., Delaney R., Lädermann A., Schwank A., Struyf F. (2023). Massive Irreparable Rotator Cuff Tears: Which Patients Will Benefit from Physiotherapy Exercise Programs? A Narrative Review. Int. J. Environ. Res. Public Health.

[20] Burks R.T., Crim J., Brown N., Fink B., Greis P.E. (2009). A prospective randomized clinical trial comparing arthroscopic single- and double-row rotator cuff repair: Magnetic resonance imaging and early clinical evaluation. Am. J. Sports Med..

[21] Lapner P., Henry P., Athwal G.S., Moktar J., McNeil D., MacDonald P. (2022). Treatment of rotator cuff tears: A systematic review and meta-analysis. J. Shoulder Elb. Surg..

[22] Jost B., Zumstein M., Pfirrmann C.W.A., Gerber C. (2006). Long-term outcome after structural failure of rotator cuff repairs. J. Bone Jt. Surg..

[23] Denard P.J., Jiwani A.Z., Lädermann A., Burkhart S.S. (2012). Long-term outcome of arthroscopic massive rotator cuff repair: The importance of double-row fixation. Arthrosc. J. Arthrosc. Relat. Surg..

[24] Jiang Q., Liu Y., Zhao Z., Chen Y., Deng Y. (2025). Association Between Preoperative Expectations and Postoperative Satisfaction in Patients Undergoing Arthroscopic Rotator Cuff Repair. J. Coll. Physicians Surg. Pak..

[25] Felsch Q., Mai V., Durchholz H., Flury M., Lenz M., Capellen C., Audigé L. (2021). Complications Within 6 Months After Arthroscopic Rotator Cuff Repair: Registry-Based Evaluation According to a Core Event Set and Severity Grading. Arthrosc. J. Arthrosc. Relat. Surg..

[26] Desai V.S., Southam B.R., Grawe B. (2018). Complications Following Arthroscopic Rotator Cuff Repair and Reconstruction. JBJS Rev..

[27] Tawfik A.M., El-Morsy A., Badran M.A. (2014). Rotator cuff disorders: How to write a surgically relevant magnetic resonance imaging report?. World J. Radiol..

[28] Talabard M.P., Regnard N.-E., Omoumi P., Texeira P.A.G., Feydy A. (2025). How Can Artificial Intelligence Help Avoid Mistakes in Musculoskeletal Imaging?. Semin. Musculoskelet. Radiol..

[29] Longo U.G., Bandini B., Mancini L., Merone M., Schena E., de Sire A., D’Hooghe P., Pecchia L., Carnevale A. (2025). Artificial Intelligence in Rotator Cuff Tear Detection: A Systematic Review of MRI-Based Models. Diagnostics.

[30] Chen C., Cui Z. (2025). Impact of AI-Assisted Diagnosis on American Patients’ Trust in and Intention to Seek Help from Health Care Professionals: Randomized, Web-Based Survey Experiment. J. Med. Internet Res..

[31] Heisinger S., Salzmann S.N., Senker W., Aspalter S., Oberndorfer J., Matzner M.P., Stienen M.N., Motov S., Huber D., Grohs J.G. (2024). ChatGPT’s Performance in Spinal Metastasis Cases—Can We Discuss Our Complex Cases with ChatGPT?. J. Clin. Med..

[32] Cestonaro C., Delicati A., Marcante B., Caenazzo L., Tozzo P. (2023). Defining medical liability when artificial intelligence is applied on diagnostic algorithms: A systematic review. Front. Med..

[33] Hasan S.S., Fury M.S., Woo J.J., Kunze K.N., Ramkumar P.N. (2025). Ethical Application of Generative Artificial Intelligence in Medicine. Arthrosc. J. Arthrosc. Relat. Surg..

[34] Günay A.E., Özer A., Yazıcı A., Sayer G. (2024). Comparison of ChatGPT versions in informing patients with rotator cuff injuries. JSES Int..

[35] Karjalainen T.V., Jain N.B., Heikkinen J., Johnston R.V., Page C.M., Buchbinder R. (2019). Surgery for rotator cuff tears. Cochrane Database Syst. Rev..

[36] Kuhn J.E., Dunn W.R., Sanders R., Baumgarten K.M., Bishop J.Y., Brophy R.H., Carey J.L., Holloway B.G., Jones G.L., Ma C.B. (2024). The Predictors of Surgery for Symptomatic, Atraumatic Full-Thickness Rotator Cuff Tears Change over Time: Ten-Year Outcomes of the MOON Shoulder Prospective Cohort. J. Bone Jt. Surg..

[37] Schairer W.W., Nwachukwu B.U., Fu M.C., Warren R.F. (2018). Risk Factors for Short-term Complications After Rotator Cuff Repair in the United States. Arthrosc. J. Arthrosc. Relat. Surg..

[38] Megafu M., Guerrero O., Yendluri A., Parsons B.O., Galatz L.M., Li X., Kelly J.D., Parisien R.L. (2025). ChatGPT and Gemini Are Not Consistently Concordant with the 2020 American Academy of Orthopaedic Surgeons Clinical Practice Guidelines When Evaluating Rotator Cuff Injury. Arthrosc. J. Arthrosc. Relat. Surg..

